# Supplementary figures and images for: The human cytomegalovirus protein UL147A downregulates the most prevalent MICA allele: MICA*008, to evade NK cell-mediated killing
Source: PLoS Pathog. 2021 May 3;17(5):e1008807. doi: 10.1371/journal.ppat.1008807 (PMC8118558; doi:10.1371/journal.ppat.1008807)

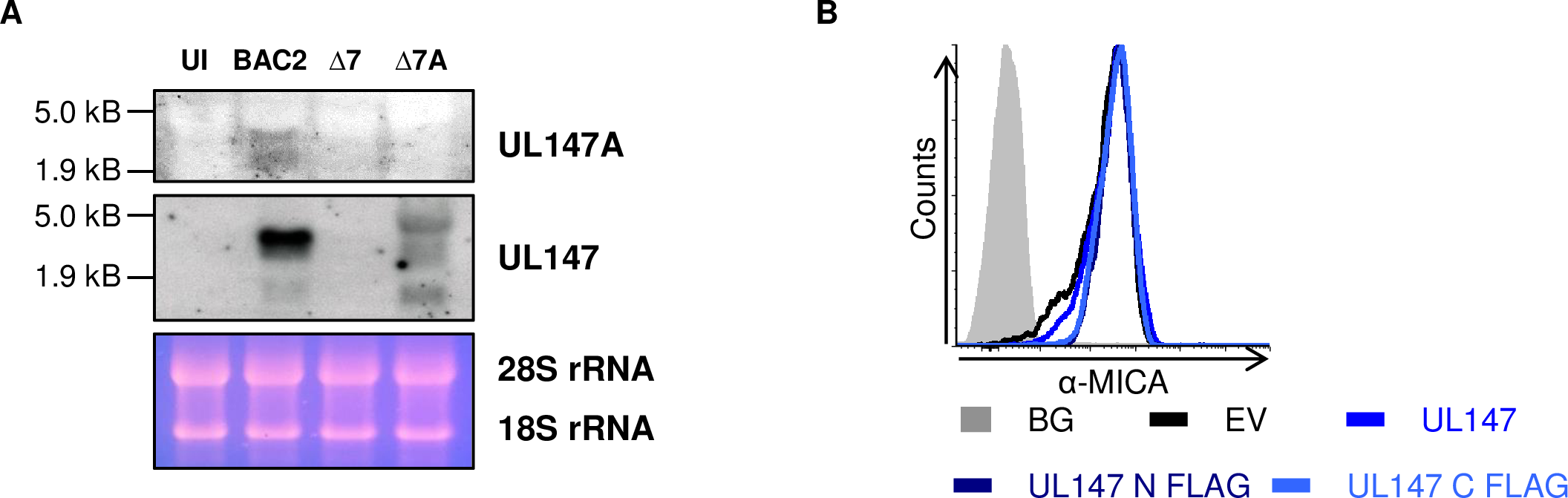

Supplement: S1 Fig — ΔUL147 deletion mutant does not express UL147A. (A) MRC-5 cells were infected with the indicated viruses. At 72 hours post infection (hpi), total RNA was isolated. Transcripts were visualized by northern blot analysis using gene-specific probes. The rRNA signals served as a loading control. (B) RKO MICA*008 cells were transduced with an EV, UL147, UL147 N-FLAG, UL147 C-FLAG. MICA surface expression was assayed by flow cytometry. Gray-filled histogram represent a secondary antibody staining of RKO MICA*008 EV cells, similar to all other control stainings. Histogram represents one of 3 biological repeats. (TIF) [file ppat.1008807.s001.tif]

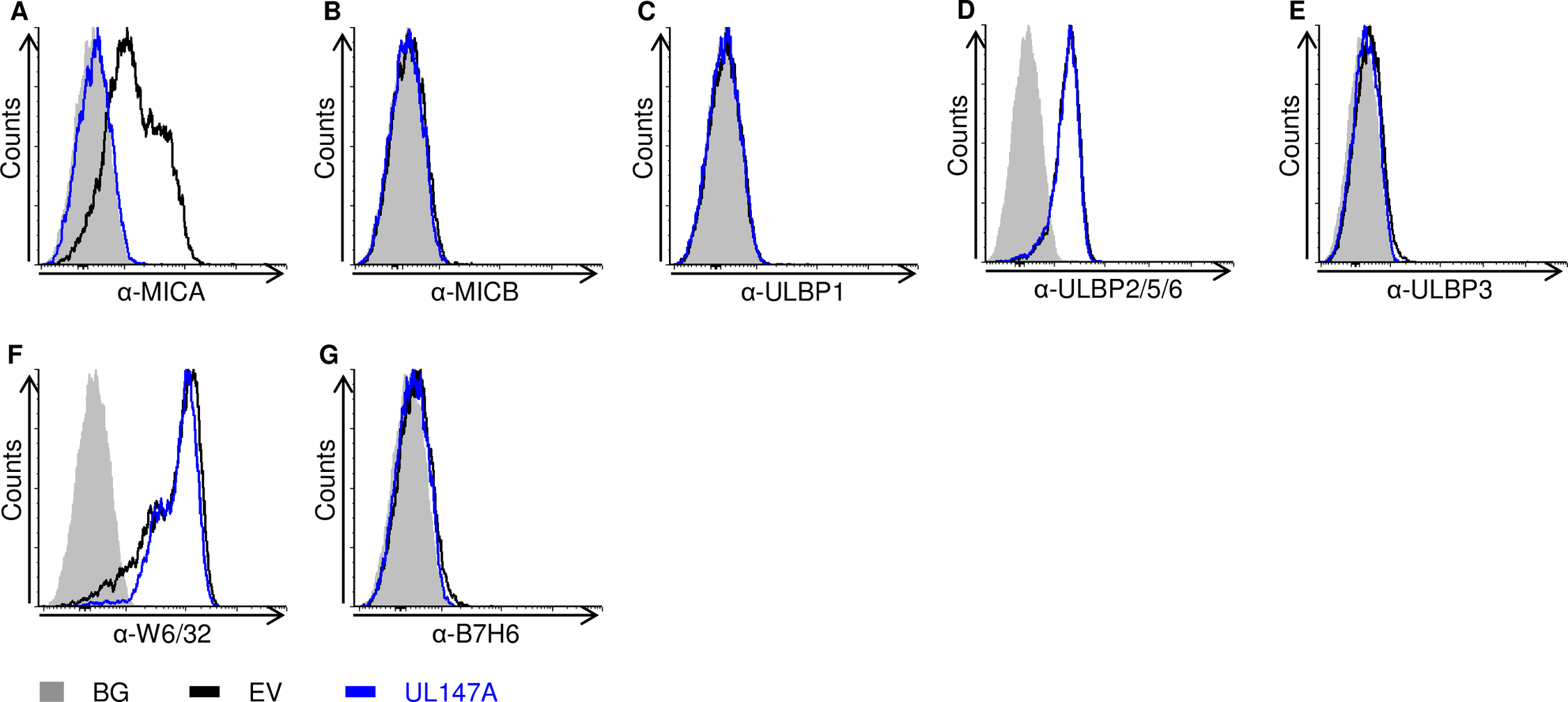

Supplement: S2 Fig — UL147A specifically targets MICA*008. FACS staining for NK ligands (indicated in the figure) of RKO MICA*008 cells transduced with an empty vector (EV; black histogram) or UL147A (blue histogram). Gray-filled histograms represent secondary antibody staining. Representative of two independent experiments. (TIF) [file ppat.1008807.s002.tif]

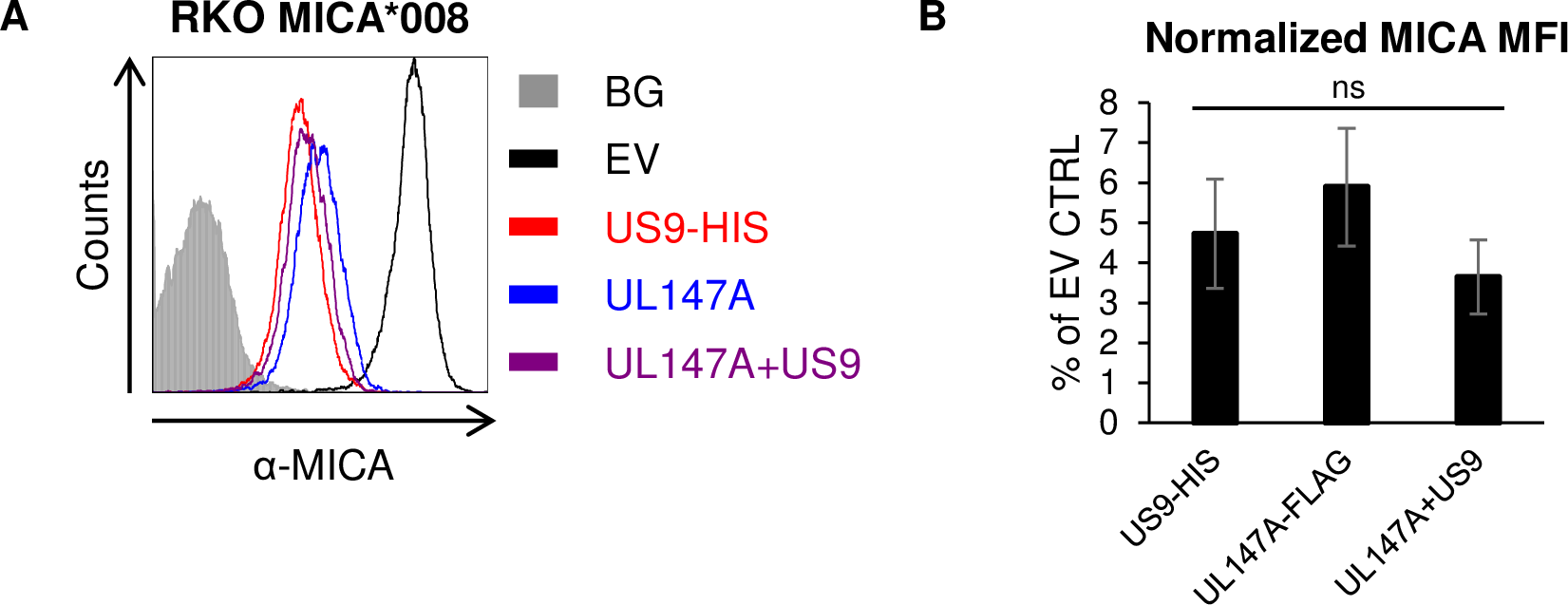

Supplement: S3 Fig — UL147A and US9 are redundant in an overexpression model. (A) RKO MICA*008-HA cells were transduced with an EV, US9-HIS, UL147A-FLAG or with US9-HIS and UL147A-FLAG together to assess synergism between the two. MICA surface expression was assayed by flow cytometry. Gray-filled histograms represent secondary antibody staining of EV cells, all control stainings were similar to the one shown. Representative of three independent experiments. (B) Quantification of MICA surface expression shown in (A), normalized to the EV control. Error bars show mean ±SEM for three independent experiments. A one-way ANOVA was performed to compare the normalized MICA median fluorescence intensity (MFI) between US9, UL147A and the two proteins together. There was no significant effect at the p<0.05 level for all conditions [F (2,6) = 0.76, p = 0.5]. Full experimental data and statistics can be found in S1 Data. (TIF) [file ppat.1008807.s003.tif]

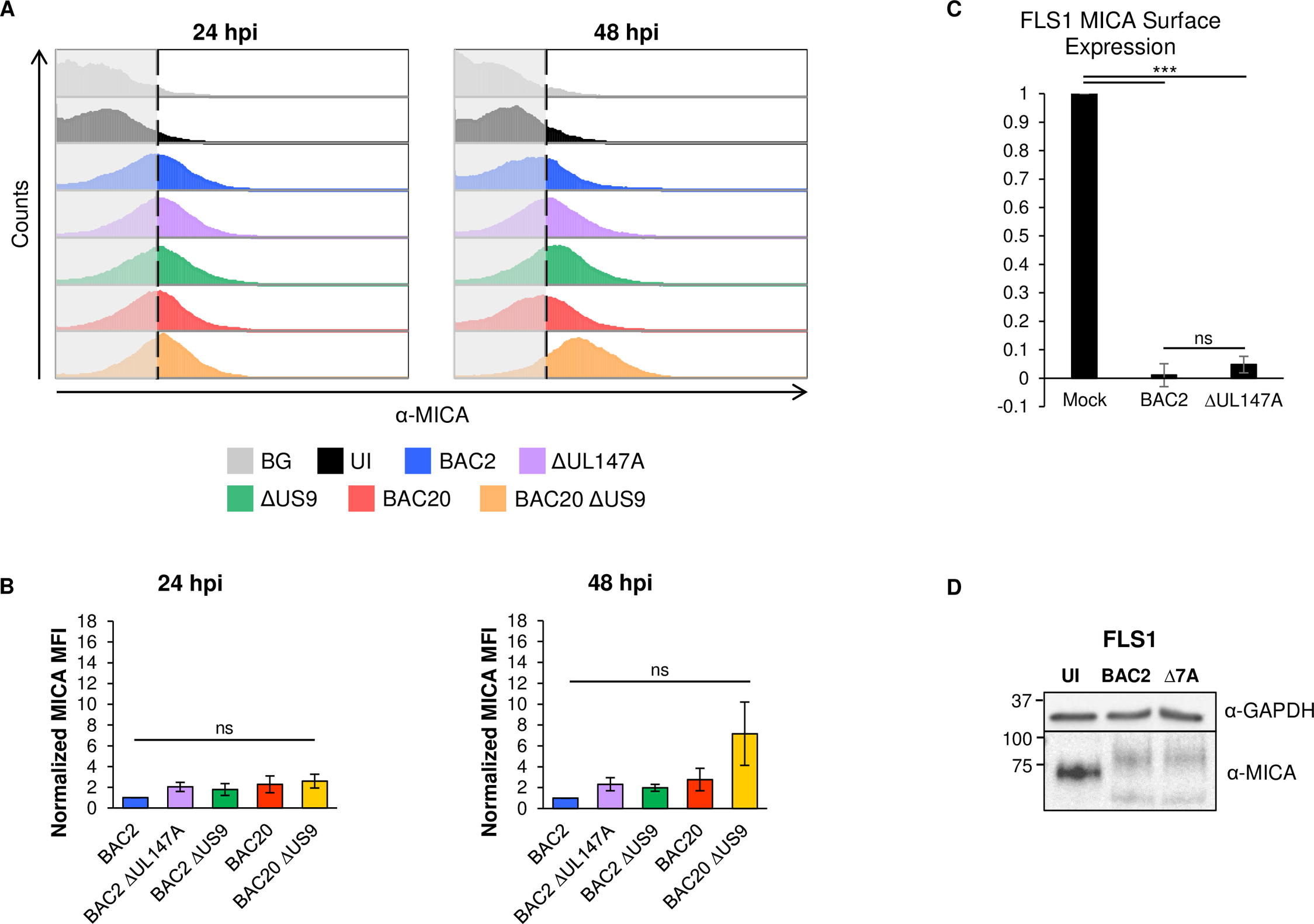

Supplement: S4 Fig — UL147A kinetics and MICA allele specificity during infection. (A-B) MRC-5 HLFs (MICA*008 homozygous) were either uninfected (UI) or infected with the indicated HCMV strains. Cells were harvested 24 or 48 hours post infection (hpi). (A) MICA surface expression was assayed by flow cytometry. Gray-filled histograms represent secondary antibody staining of EV cells, all control stainings were similar to the one shown. (B) MICA median fluorescent intensity (MFI) values shown in (A) were quantitated and normalized to BAC2-infected cells. Data show mean ±SEM of three independent experiments. A one way ANOVA was performed. There was no significant effect at the p<0.05 level for all conditions for 24 hpi [F (4,10) = 1.15, p = 0.38] or for 48 hpi [F (4,10) = 2.63, p = 0.097]. ns not significant. (C-D) FLS1 HFFs (endogenous full length MICA*004/*009:01-*049) were either uninfected (UI) or infected with the indicated HCMV strains. Cells were harvested 72 hpi. (C) MICA surface expression was assayed by flow cytometry and the MFI values of three independent experiments were normalized to uninfected cells. A one-way ANOVA was performed with a significant effect at the p<0.05 level for all conditions [F (2,6) = 377.51, p = 4.9∙10−7], followed by a post-hoc Tukey test. *** p < 0.001, ns not significant. (D) Cells were lysed and a western blot was performed using anti-MICA antibody for detection of MICA, and anti-GAPDH antibody as a loading control. Representative of two independent experiments. Full experimental data and statistics can be found in S1 Data. (TIF) [file ppat.1008807.s004.tif]

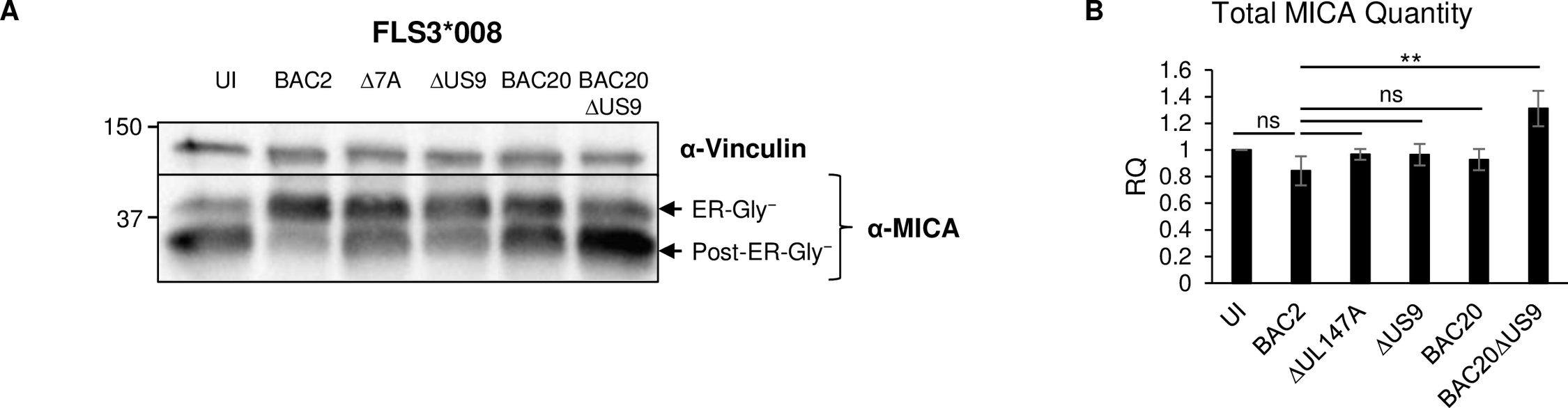

Supplement: S5 Fig — UL147A and US9 induce maturation arrest but also reduce MICA*008 quantity during HCMV infection in FLS3*008 cells. FLS3*008 HFFs (overexpressing MICA*008) were either uninfected (UI) or infected with the indicated HCMV strains. Cells were harvested at 72 hr post infection (hpi) and lysates were prepared (as part of the same experiment shown in Fig 5F). (A) Lysates were deglycosylated with PNGaseF and a western blot was performed using anti-MICA antibody for detection of MICA and anti-vinculin antibody as a loading control. (B) Quantification of deglycosylated MICA*008 forms shown in (A). MICA levels were quantified relative to the loading control. RQ, relative quantification. Data show mean ±SEM for three independent experiments. A one-way ANOVA was performed with a significant effect at the p<0.05 level for all conditions [F (5,12) = 3.51, p = 0.035]. A post-hoc Dunnett’s test was used to compare BAC2 MICA protein levels to each infected cell. ** p < 0.01, ns not significant. Full experimental data and statistics can be found in S1 Data. (TIF) [file ppat.1008807.s005.tif]

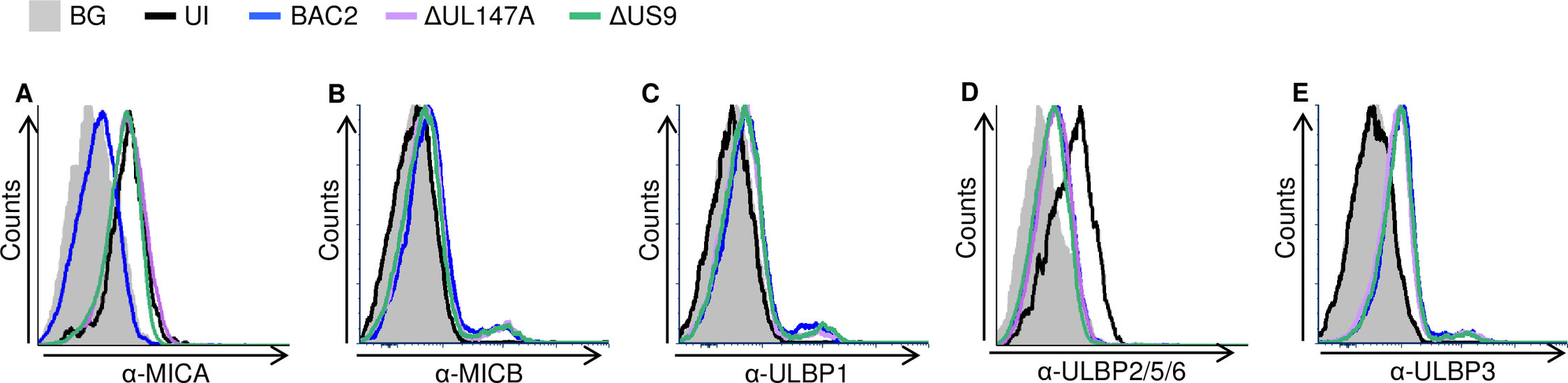

Supplement: S6 Fig — UL147A-deficient and US9-deficient HCMV mutants are impaired in MICA*008 downregulation only, among NKG2D ligands. (A-E) MRC-5 HLFs (MICA*008 homozygous) were either uninfected (UI) or infected with the indicated HCMV strains. Cells were harvested 72 hours post infection (hpi). Surface expression of NKG2D ligands was assayed by flow cytometry: MICA (A), MICB (B), ULBP1 (C), ULBP2/5/6 (D), ULBP3 (E). Gray-filled histograms represent a background staining of uninfected cells, similar to all other cells. Representative of two independent experiments. (TIF) [file ppat.1008807.s006.tif]
