# Supplementary material for: The human cytomegalovirus protein UL147A downregulates the most prevalent MICA allele: MICA*008, to evade NK cell-mediated killing
Source: PLoS Pathog. 2021 May 3;17(5):e1008807. doi: 10.1371/journal.ppat.1008807 (PMC8118558; doi:10.1371/journal.ppat.1008807)
Supplement: Attachment — Submitted filename: point by point.docx [file ppat.1008807.s008.docx]

Reviewer's Responses to Questions

**Part I - Summary**
Please use this section to discuss strengths/weaknesses of study, novelty/significance, general execution and scholarship.

Reviewer #1: Seidel and colleagues describe a role for HCMV UL147A in inhibiting NK cells through the proteasomal degradation of MICA*008, a ligand for NKG2D. To date, a function has not been ascribed to UL147A, while MICA*008 is unusual in that it is a MICA allele with a GPI anchor. UL147A is thus the second HCMV gene that has been shown to target MICA*008. The manuscript also describes mechanistic expts showing that UL147A binds MICA*008 and UL147A function is dependent on the non-canonical system of GPI anchoring specific for MICA*008. The data presented in the main text is convincing as far as it goes, but there are a number of points, addressing of which would give a better physiological context to their data and its interpretation.

We thank the reviewer for the positive overall assessment of our work. We added additional experiments as requested by the reviewer, and feel confident that we now address the reviewer’s concerns.

Reviewer #2: Seidel et al report another HCMV gene that is able to interfere with the normal expression of MICA*008, an NKG2D ligand which would normally lead to NK cell activation. HCMV is notable for having multiple different viral proteins that interfere with the multiple different human NKG2D ligands and in the case of MICA multiple different alleles. The prevalent MICA allele 008 has some unique features and this group have previously demonstrated that Us9 is able to downregulate it. However, downregulation is incomplete which has led to the current work.

Results are shown with various HCMV strains with a combination of block and single gene deletions the results of which suggest that the ULb’ gene UL147A is also able to specifically target MICA*008. A standard but entirely acceptable set of experiments then show that UL147A is sufficient in an overexpression system, that the protein is degraded, UL147A is ER resident and that UL147A-MICA*008 interact and target MICA*008 for proteasomal degradation. Alterations of MICA*008 domains into MICA*004 show that frameshift and STOP codons are required and transfer of the 008 TM into MICB subsequently allows UL147A targeting.

The requirement for what appears redundant Us9 and UL147A control of MICA*008 is also addressed suggesting that greater levels of MICA*008 are induced in a double negative. A functional NK cell killing assays also show that deletion of either increases killing, however the increased MICA*008 levels with a double KO is not investigated in this functional assay.

The work presented has been well performed and controlled, the data is very clear and well presented, the paper is well written and mostly clear to follow. The targeting NKG2DL for down regulation by a viral gene and targeting to proteosomal degradation is not novel, nor that HCMV has multiple genes that target important immune functions, nor that MICA*008 can be targeted by HCMV. However, the work does convincingly identify another HCMV gene that does this and the mechanism and as such adds to the knowledge about NK cell cell evasion by this virus.

We thank the reviewer for the kind remarks about the quality of our work. We could not use the current double KO in killing assays due to the fact that it lacks several additional HCMV immunoevasins and thus the comparison between it and the single mutants would not be valid. Due to technical reasons, we were unable to generate a double mutant using the same BAC as the single mutants. We now address this point in the discussion.

Reviewer #3: The manuscript entitled "The human cytomegalovirus protein UL147A downregulates the most prevalent MICA allele: MICA*008, to evade NK cell-mediated killing" submitted to PLoS Pathogens for publication, explores the contribution of a previously poorly characterized viral gene product on the surface expression of a relatively common MICA allele. Many studies have confirmed that genes non-essential for viral growth are often lost through in vitro passage, yet these serve critical functions in evading the host immune response. UL147A, with is found within the ULb’ region of the HCMV genome, is suggested to play a similar role. It is hypothesized that evasion of NK cell mediate killing that naturally occurs following recognition of MICA*008 is avoided if the virus can prevent its surface expression. The authors of this manuscript have performed a series of elegant biochemical experiments to fully characterize the extent and specificity and mechanism of MICA*008 downregulation by UL147A. The work overall is of high significance to the field of HCMV biology yet the impact to NK biology is less well-developed. However, the data support the authors’ claims, although the overall message of NK cell immune evasion may not be the most accurate description of this work.

This manuscript initially demonstrates how viral mutants downregulate MICA*008 to varying degrees, followed by biochemical studies to quantify and characterize the apparent mechanism. Furthermore, microscopy qualitatively confirms altered distribution of MICA*008 that is due to UL147A expression. Interestingly, the effect of UL147A is only to the MICA*008 allele, and the authors perform a thorough set of experiments, with chimeric constructs, to understand how the non-canonical maturation pathway play a role. Finally, the authors end with an in vitro assay to characterize increased NK cell killing of virus-infected cells deficient in UL147A. However, the reversal of the phenotype of killing is not complete, and the title therefore may be misleading.

Overall, the authors perform the appropriate experiments to assess the biological role of UL147A and it’s effect on MICA*008. A few outstanding questions remain and have been listed below. However, my concern is that both the title and the abstract suggest the that NK cell evasion is a major focus of the manuscript; unfortunately from my perspective it is not. More descriptive information relative to the paper’s findings should be included in the abstract, with slightly less emphasis on NK cell evasion, proportional to the data presented.

We thank the reviewer for the positive comments on our manuscript.

Regarding the reviewer’s questions about the focus of our work, we agree that NK biology is not the focus of our work. However, we consider that characterization NK cell ligand manipulation falls under the category of NK cell evasion, even if most of the study focuses on the ligand itself and not on its interactions with the receptor on NK cells. This is the commonly held view in this field. For instance, similar manuscripts describing HCMV proteins that target NK ligands bear titles such as “Two Novel Human Cytomegalovirus NK Cell Evasion Functions Target MICA for Lysosomal Degradation” (Fielding et al, PLoS Pathogens, 2014).

In light of the reviewer’s comments, we modified the abstract with less emphasis on NK cell biology.

Regarding the reviewer’s comment about incomplete reversal of reduced killing in the deletion mutants. This comment refers to a previous version of the killing experiment. In the current version (Fig. 7), we show the results from several different donors, showing variable killing of the UI cells, resulting in partial or full restoration of killing levels. However, we consider this comparison to be uninformative for several reasons:

- Firstly, the variability of UI cells killing between NK donors, presumably due to donor intrinsic factor such as mismatching of HLA alleles, receptor polymorphisms etc.
- Secondly, as cellular response to infection is to upregulate NK activating ligands, among them MICA, we would have expected to see increased killing of infected cells but for the activity of immune evasion mechanisms. Since there are multiple other HCMV immunoevasins, no single deletion can restore killing levels to their full potential value. What we observe is an intermediate value determined by the equilibrium between cellular responses and viral countermeasures, that may or may not incidentally resemble the killing of UI cells.

For this reason, in Fig. 7 we focus on the effect of the deletions compared to the parental virus and explain this point in the manuscript.

**Part II – Major Issues: Key Experiments Required for Acceptance**
Please use this section to detail the key new experiments or modifications of existing experiments that should be absolutely required to validate study conclusions.

Generally, there should be no more than 3 such required experiments or major modifications for a "Major Revision" recommendation. If more than 3 experiments are necessary to validate the study conclusions, then you are encouraged to recommend "Reject".

Reviewer #1: Broad Points
i. The physiological interpretation of what happens during a HCMV infection cannot be concluded from the data. More detailed points are given below, but broadly, none of the viruses used are wildtype HCMVs, while many of the experiments are performed only using ectopic expression rather than in the context of a HCMV infection.

BAC-cloned viruses are commonly used and well established for the research of HCMV. Specifically, BAC2/AD169VarL clones that are close to WT in their complement of genes have been studied and used extensively in research (VTK Le et al., JVI 2011; Yamin et al. Cell Reports 2016; Dassa et al., JVI 2018; VTK Le-Trilling et al., Cell Reports 2020). We previously compared BAC2/AD169VarL to TB40/E, a strain which does not harbor large deletions and is considered close to clinical, and found no differences in the magnitude of their effect on MICA*008 (Seidel et al., Cell Reports 2015, Fig. S5B).

As reviewer #1 suggested, we validated our results from the overexpression system also in the context of infection, as described below.

ii. The NK assays are preliminary.

We repeated the NK assays using several different NK donors and also during blocking of the NKG2D receptor, and performed statistical analysis of the pooled data set (revised figure 7).

Detailed points
i. Figure 1 shows a screen of HCMVs and deletion mutants to identify UL147A as targeting MICA*008 and clearly shows this. However there is no inclusion of a virus with a complete UL/b’ region (AD169VarL is missing UL140-144). The authors say they ascertained which gene(s) in the UL/b’ region were responsible, but they haven’t screened the whole region, especially as UL142 also is known to target MICA alleles. This is perhaps of greater significance as the shift caused by AD169VarS looks larger than the shifts by the BAC2 (AD169VarL) mutants.

To address the reviewer’s concerns, we repeated the screen in Figure 1 with repeats and performed a statistical analysis of the independent experiments. Effect size in VarS/BAC20 was not significantly different from the block deletion mutants that lack UL147A or from ∆UL147A itself. In figure 5, we further validate the equivalence of BAC20 and ∆UL147A in surface expression and overall protein quantity of MICA*008. Regarding UL142, it has been previously shown to have no effect on the MICA*008 allele (Ashiru et al, 2009) and it is not certain that it even affects full length MICA (Fielding et al, 2014). We previously compared BAC2/VarL and TB40/E infected cells and observed no significant differences in MICA*008 levels (Seidel et al., 2015), rendering it unlikely that the UL140-144 region mediates significant effects on MICA*008.

ii. Figure 1D, F – the shifts are small so an idea of variation through replicates and a statistical test would be stronger rather than a bar chart repeating the flow histogram data.

We repeated this experiment and now show statistical analysis for at least three independent repeats per mutant (Fig. 1), with more robust effect size.

iii. Figure 2 – This is solid data of the impact of UL147A when ectopically expressed at high level, but what happens during a HCMV infection? Experiments comparing ∆UL147A with parent HCMV should be provided.

As suggested, further infection experiments were performed and are described in the revised Fig. 5-7 and Fig. S4,5, including validation of UL147A MICA*008 allele specificity in the context of HCMV infection, as well as western blot assays.

iv. Figure 3 – again these experiments are very clean but do not provide information on events during a HCMV infection. UL147A should be tagged in the virus and data provided during a HCMV infection.

As suggested, further infection experiments were performed including CHX chase experiments. Regarding IF in the infected cells, we generated a recombinant virus with FLAG-tagged UL147A (UL147A R) on the BAC2 background – see below flow cytometry of MICA surface expression with statistics of 3 repeats, and western blot analysis all demonstrating the tagged-UL147A behaves the same as the parental BAC2 virus.

However, we were unable to complete immunofluorescence experiments during infection due to technical difficulties. Given the considerable delays in submitting a corrected manuscript, we decided not to delay submission until these difficulties could be resolved. If, however, the reviewer considers this to be a crucial point, we will be able to add it.

v. Figure S2 – Data showing a direct interaction between UL147A and MICA*008 is important and should be provided in the main text if the Figure can be reproduced in a convincing manner (perhaps in the presence of a proteasome inhibitor?). This should also be performed in the context of a HCMV infection.

We attempted to reproduce this result as requested, both in an overexpression system and during HCMV infection utilized the FLAG-tagged UL147A described above, but received weak and inconsistent signal. We are not sure whether this is due to transient/weak/low prevalence interactions between MICA*008 and UL147A, or that such interactions do not exist and are an artifact, or whether it is due to technical difficulties with the assay. While we did employ proteasome inhibitors, the slow degradation kinetics of MICA*008 require prolonged treatment times to attain effects, and these are not well tolerated by the cells, limiting their efficacy.

We recently showed that the some US9 domains mediate MICA*008 degradation without direct interactions with it, so it is entirely possible that UL147A does not interact with MICA*008. Other US9 domains did interact with MICA*008, and knockdown of the E3-ligase complex that mediates MICA*008 degradation stabilized the interactions and improved IP results more significantly than proteasome inhibition (Seidel et al., Nature Communications, in press).

This strategy requires the characterization of UL147A-interacting E3 ligase complexes (if such exist), and their knockdown. While we do intend to identify UL147A‘s cellular interaction partners (whether or not they include MICA*008), given the challenges outlined above, we believe that this is beyond the scope of the current manuscript. Therefore, we removed this figure entirely from the manuscript.

vi. Figure 5 – The data does not link the effect back to the targeted gene and HCMV genes are multifunctional. An expt with anti-MICA mAb is important to show that it is specifically the effect of knocking out UL147A (or US9) on MICA*008 that causes the increase in susceptibility to NK cells. It may also provide some explanation for the lack of correlation between killing of mock targets and HCMV-infected ones – mocks have hardly any MICA*008 at 72hrs and are killed even more than the BAC2 mutants. A greater range of donors should also be provided allowing statistical testing.

We agree with the reviewer that the suggested additions to the killing experiment are of high importance for the support of our claims.
We have previously searched for an anti-MICA blocking antibody with no success, and others had similar difficulties (Fielding et al., PLoS Pathogens, 2014). However, as MICA is an NKG2D ligand, we decided to use an anti-NKG2D blocking antibody instead. To verify that this is an acceptable substitute, we stained against all the NKG2D ligands during infection with the relevant HCMV mutants, and indeed saw that they all had a similar effect on each of the ligands, and only MICA*008 was differentially regulated (Fig. S5). Killing assays were repeated with more NK donors, allowing statistical analysis. These data are presented in revised Fig. 7.

Reviewer #2: (No Response)

Reviewer #3: What is the effect of deleting UL147A on viral growth in vitro? I would suggest performing a multi-step growth curve to show that viral viability is not affected. Maybe UL147A has an additional role in viral replication and thus the partial effect of NK cell killing seen in Figure 5 is due to low viral titers per cell?

We previously verified that BAC2 and BAC20 yield similar virus titers during infection, over the course of 9 days post infection (Le-Trilling et al. 2020). As BAC20 lacks the UL*b’* region, including UL147A, these data are sufficient to show that this region, and all the genes in it, are dispensable for viral replication in fibroblasts. Other groups reported similar findings for Ul*b’* genes, which are frequently lost or mutated during serial passage of HCMV in fibroblasts (see for example: Cha et al., JVI 1996 doi: 10.1128/JVI.70.1.78-83.1996).

Further, we validated infection efficacy in all our experiments. Samples were used only if >75% infected and if the variance between strains was <15% (as detailed in the materials and methods section). Therefore, we think it is highly unlikely our results stem from differential HCMV infection.

Regarding the reviewer’s comment about incomplete reversal of reduced killing in the deletion mutants, it refers to a previous version of the killing experiment. In the current version (revised Fig. 7), we demonstrate that levels of killing in the UI control vary between donors, sometimes leading to full “restoration” and sometimes to partial “restoration”.

It should be noted that HCMV has many other viral immunoevasins that reduce NK mediated killing, even in the absence of MICA*008-targeting proteins. Therefore, the comparison of killing levels between UI and infected cells is uninformative, since thse killing of infected cells represent a new balance between cellular responses such as induction of stress induced ligands on the one hand, and viral countermeasures on the other hand. The more relevant comparison is between parental and mutant-infected cells. We hope the revised version of this figure and the text accompanying it addresses the reviewer’s concerns.

**Part III – Minor Issues: Editorial and Data Presentation Modifications**
Please use this section for editorial suggestions as well as relatively minor modifications of existing data that would enhance clarity.

Reviewer #1: i. MFI is not defined in the Figure legends (median or standard mean or geometric mean?).

Clarification was added to the figure legends.

ii. Figure 5 - Much more detail is required in the methods – are these NK lines or ex vivo NK cells isolated from PBMC?

Description of the NK cell isolation (from PBMCs) is included in the “cells” section of the Materials and methods. Clarification was added to the “NK cell killing assay” section as well.

Reviewer #2: Results for Fig 1
While reading the figure, given that this group have already described Us9 to have MICA*008 downregulation function I was surprised hat the virus strains used all express Us9 and did wonder what a mutant with both would look like. Clearly, this was addressed in experiments detailed in Figure 5. For clarity the authors might like to add a statement that explains why the exps are done in a US9+ background and that this will be addressed later. Likewise, the abstract/summary does not mention the Us9/UL147A double mutant.

We agree that the use of ∆US9 deletion background would have increased the signal of other MICA*008-targeting proteins. However, we began the search with reagents we had available, and since we observed a significant signal despite the presence of US9, generation of a mutant library on a ∆US9 background was not necessary. We added a clarification to the manuscript.

FigS2 demonstrates co-IP data for MICA*008-UL147A I am unsure why it needs to be a supplement and is not panel D in Figure 3 as it completes that analysis and is not really supplemental.

We attempted to reproduce this result as requested, both in an overexpression system and during HCMV infection utilized a recombinant virus with FLAG-tagged UL147A, but received weak and inconsistent signal. We therefore decided to remove this experiment from the manuscript, until we can more fully address the question of UL147A’s cellular interaction partners in future studies.

The data shown I Fig5 addresses Us9 and UL147A redundancy and combined function and clearly in a HCMV infection model removal of both via the BAC20 (VarS AD169 short ULb’) delta Us9 construct shows the highest MICA*008 accumulation. Effect on NK cell killing is also addressed, however a double US9 UL147A mutant is not used, I appreciate that delta Us9 on a BAC20 background also deletes other NK evasion genes and would not be suitable for the analysis, but the double mutant could have been made on the BAC2 background to address this? Would the authors predict a further increase in cytotoxicity or not? Figure 5 utilizes a number of BAC HCMV backgrounds as well as additional gene KO the authors should consider adding a little more information to the key to aid the reader – BAC2 is VarL full length Ulb’ while BAC20 is VarS and is deleted for UL147A (as well as most of Ulb’).

We agree with the reviewer that a double mutant on the BAC2 background would have been preferable. However we were not able to generate such a mutant due to technical difficulties, and since BAC20 and ∆UL147A are equivalent in their effect on MICA*008, this is good enough for all other purposes. Furthermore, such a double deletion would not necessarily have increased NK mediated killing beyond the effect seen for each single mutant, since NK activation is governed by integration of stimulatory and inhibitory signals, with threshold effects. Indeed, double deletion of the US18 and US20 immunoevasins that target full length MICA alleles increased killing only mildly compared to each single mutant (Fielding et al., 2014). We now address this point in the discussion.

We thank the reviewer for his suggestion regarding adding more information to Fig. 5 and have now added visual representation of UL147A and US9 presence in panels C and F of the revised figure.

Reviewer #3:

1. Since NK cell evasion is a major focus of the paper, why is there no mention in the discussion section of how these findings compare and contrast with similar works?

We addressed this issue and extended the discussion, comparing US9 and UL147A’s effect to the joint effects of US18 and US20 on full length MICA alleles.

2. In Figure 1, were VH3 HFFs infected with UL147A deficient HCMV? How does down-regulation compare to VarS?

Figure 1 shows the original discovery experiments conducted in VH3 fibroblasts to check for MICA*008-targeting genes in the ULb’ region, by comparing VarS (equivalent to BAC20, this is the original, non-BAC cloned strain) with BAC2 (Which is BAC-cloned VarL) infection. As such, BarS lacks the Ulb’ region and all genes therein, including UL147A.

BAC20 and the individual deletion mutants including ∆UL147A were generated later and used in the more extensive screen conducted in MRC-5 HLFs. We modified the text to clarify this point.

3. In Figure 1, there are no error bars on the bar plots (D,F). Were these experiments performed only once? There is no mention of replicates in the legend or text.

We repeated this experiment and now show statistical analysis for at least three independent repeats per mutant.

4. Similarly for Figure 2, S2, and S3, there is no mention of replicate analysis.

We repeated the relevant experiments and stated the number of replicates for each experiment in the legend. When data was quantified, a relevant statistical analysis was performed. As for Fig. S2, repeating these experiments yielded inconsistent results and therefore excluded it from the manuscript.

5. In Figure 5, red colored bars are not universally applied in the first and second panels of (B) as stated in the legend.

The figure and figure legend were revised and corrected, and these panels can now be found in Fig. S4.
